# Interleukin 1-****β****, Interleukin-1 Receptor Antagonist, and Interleukin 18 in Children with Acute Spontaneous Urticaria

**DOI:** 10.1155/2013/605262

**Published:** 2013-12-29

**Authors:** E. Machura, M. Szczepańska, B. Mazur, M. Barć-Czarnecka, A. Kasperska-Zając

**Affiliations:** ^1^Department of Pediatrics, Medical University of Silesia, Ulica 3-go Maja 13-15, 41-800 Zabrze, Poland; ^2^Department of Microbiology and Immunology, Medical University of Silesia, Ulica Jordana 19, 41-808 Zabrze, Poland; ^3^Chair and Clinical Department of Internal Diseases, Dermatology and Allergology, Medical University of Silesia, Ulica M. Curie-Skłodowskiej 10, 41-800 Zabrze, Poland

## Abstract

Very little is known about the role of interleukin-1**β** (IL-1**β**) and interleukin-18 (IL-18) in urticaria. *Material and Methods*. Serum levels of IL-1**β**, IL-1 receptor antagonist (IL-1RA), and IL-18 were measured in 56 children with urticaria and in 41 healthy subjects. *Results*. Serum IL-1**β** did not differ between children with acute urticaria and controls. Children with single episode of urticaria had higher levels of IL-1RA and IL-18 than healthy subjects. In children with single episode of urticaria, level of IL-1RA correlated with C-reactive protein (CRP), D-dimer, and IL-1**β** levels. In subjects with recurrence of urticaria IL-1RA was positively correlated with WBC and D-dimer levels. No correlation of cytokine levels and urticaria severity scores (UAS) in all children with urticaria was observed. In children with single episode of urticaria UAS correlated with CRP level. In the group with single episode of urticaria and in children with symptoms of upper respiratory infection, IL-1RA and IL-18 levels were higher than in controls. The former was higher than in noninfected children with urticaria. In conclusion, this preliminary study documents that serum IL-1RA and IL-18 levels are increased in some children with acute urticaria. However further studies are necessary to define a pathogenic role of IL-1**β**, IL-1RA, and IL-18 in urticaria.

## 1. Introduction

Urticaria is a commonly recognized skin disorder characterized by the sudden development of pruritic wheals and/or angioedema. During recent years significant progress has been made in elucidation of both the etiology and pathogenesis of urtricaria; however, data on urticaria in children still remain scarce and are mostly derived from extrapolating information obtained in adults [1]. Although pediatric urticaria has a specific clinical appearance, its pathogenesis remains partially unknown. The most common manifestation in children is acute spontaneous urticaria, affecting 4.5–15% of this age group [2]. Infections are the main trigger factor of acute urticaria in the pediatric age group. Allergy, drugs, and physical factors have been implicated in pathogenesis to a lesser extent [3]. About 20–30% of cases of acute urticaria progress to chronic and recurrent urticaria [1].

In patients with acute urticaria independently on the age, degranulation of cutaneous mast cells by a variety of causes results in the release of histamine and other inflammatory mediators which initiate local or generalized skin involvement. The increase in circulating levels of general markers of inflammation in association with the alteration of cytokine profile has been widely documented in adult patients with urticaria [4–11].

It has been described in recent literature that broad spectrum of cytokines and chemokines, including interleukin 1-*β* (IL-1*β*) and interleukin 18 (IL-18) has been involved in activation or modulation of the mast cells function [12, 13].

The IL-1 family includes 11 known members together with IL-1*α* and IL-1*β* which were discovered first. IL-1*β* and IL-18, are important representatives of this family, inducing expression of an array of various proinflammatory genes and play a central role in innate and acquired immune response [14, 15]. IL-1*β* is produced predominately in blood monocytes, macrophages, and dendritic cells. IL-1*β* promotes T-cell survival, upregulates the IL-2R on lymphocyte surface, stimulates B-lymphocytes for proliferation and antibody production, and enhances Th17 lymphocytes differentiation [14, 16].

The main source of IL-18 are monocytes and macrophages, dendritic cells and also keratinocytes, and Langerhans' cells and B-lymphocytes. IL-18 exerts a Th1 inflammatory response with simultaneous costimulation with IL-12, IL-15, or IL-2 and Th2 response without the influence of IL-12 and IL-23. IL-18 also promotes allergic inflammation through the activation of IL-4, IL-13, histamine release, and enhanced IgE production [12, 17–19].

Both IL-1*β* and IL-18 require the intracellular cysteine protease—caspase-1 for biological activity. Caspase-1 activation is mediated by a cytosolic multiprotein oligomer called the inflammasome [20]. The activity of the inflammasome is triggered not only by microbial infection, but also by a wide range of noninfectious both exogenous and endogenous stimuli. The dysregulation of inflammasome activity is associated with numerous proinflammatory and nonmicrobial diseases in humans [21, 22]. IL-1*β* and IL-18 are the pivotal cytokines in autoinflammatory diseases such as cryopyrin-associated periodic syndromes (CAPS) group and Schnitzler syndrome (SchS) which are regularly associated with urticarial rash [23]. Recent findings also suggest that both inflammasome dependent cytokines are implicated in allergic diseases such as asthma, atopic and contact dermatitis, and some forms of chronic urticaria [23–27].

The IL-1 receptor antagonist (IL-1RA), another member of IL-1 family, competitively blocks the corresponding receptors reducing the inflammation response but not exerting its own agonists activity. The balance between IL-1 and its receptor IL-1RA plays an important role in a modulation of the course of different diseases including diabetes, overweight, chronic rheumatic diseases, sepsis, colitis, and granulomatous pulmonary disease [28].

The possible role of IL-1*β* and IL-18 in acute spontaneous urticaria has not been studied in children yet. Thus, the aim of our study was to analyze serum levels of IL-1*β* and its competitive antagonist, IL-1RA and IL-18 in children with acute spontaneous urticaria.

## 2. Material and Methods

The study group consisted of 56 children (mean age 9.2 ± 1.31, range: 3.5–17 y.) consecutively referred to the pediatric clinic with a diagnosis of urticaria. Urticaria was diagnosed and classified according to EAACI/GA^2^LEN/EDF/WAO guidelines [29]. Among the study patients 39 children were classified as having single episode of acute urticaria. In remaining 17 patients urticaria occurred intermittently, but recurrently over months or years and they were classified as suffering from recurrent acute urticaria (the duration of symptom-free periods was longer than 6 weeks).

Children with physical urticaria were excluded. In all cases, any known causes of urticaria were ruled out by appropriate investigations. Each patient underwent the following laboratory tests: full blood count, urine analysis, hepatic function tests, and serum C-reactive protein (CRP) concentration. In addition, stool examination for parasites and the levels of IgA, IgM, IgG, IgE, and D-dimer were measured. In patients with recurrent urticaria the following tests were performed: *H. pylori* antigen evaluation, hepatitis serology (anti-HCV antibodies, HBsAg), rheumatoid factor, antinuclear and antithyroid microsomal antibodies, and thyroid function tests (thyroid stimulating hormone-TSH and free thyroxine-fT4). Skin prick tests and/or specific (s) IgE were performed in patients with a suggestive history for allergy. In spite of extensive studies, no cause was found in 23 patients with urticaria (acute urticaria—13 children and recurrent urticaria—10 children). The identified possible causes of urticaria were the following: upper respiratory infection (twenty one patients), food allergy (two), lambliasis (two), and drug allergy (one)—in children with acute urticaria and recurrent upper respiratory infection (five), lambliasis (one), and *H. pylori* infection (one)—in patients with recurrent urticaria. Daily urticaria severity scores (UAS) were measured through the assessment of wheals and itching on a scale 0–3 (the sum of scores: 0–6). A daily UAS of 0–3 was considered to represent mild urticaria, 3-4 moderate and 5-6 severe urticaria symptoms. IL-1*β*, IL-1RA, and IL-18 were examined in the whole urticaria group and separately in children with the clinical sings of infection and children free from infection. The control group consisted of 41 healthy children (mean age 9.4 ± 1.57, range: 2–17 y.) with a negative history of allergic disease, a normal level of total serum IgE and negative results of skin prick test to a panel of aeroallergens (dust mite, mixed grass or tree pollen, cat, and dog; Allergopharma, Reinbek, Germany). Children included into the control group attended the outpatient pediatric clinic for nonimmunological, noninflammatory health problems and needed venous puncture. The present study was approved by the Ethics Committee of the Medical University of Silesia in Katowice and written informed consent was obtained from children's parents.

### 2.1. Laboratory Measurements

#### 2.1.1. IL-1*β*, IL-1RA, and IL-18

The serum concentration of IL-1*β*/IL-1F2, IL-RA/1F3, and IL-18 was measured with an enzyme-linked immunoabsorbent assay (ELISA) using Quantikine kits (R&D Systems, Minneapolis, USA) according to the manufacturer's recommendations. Peripheral venous blood was collected into 5 mL citrate collection tubes. The collected samples were centrifuged for 15 minutes at 1000 ×g. The serum samples were divided into aliquots and stored at −80°C until assayed. We did not use the samples, which were re-thawed. The lower limits for IL-1*β*, IL-1RA, and IL-18 were 1 pg/mL, 18.3 pg/mL, and 12.5 pg/mL, respectively.

#### 2.1.2. Other Laboratory Investigations

The serum concentration of total IgE was measured by ELISA using a commercial kit (Allergopharma, Reinbeck, Germany) according to the manufacturers' instructions. The blood eosinophil counts were determined using an automatic hematologic analyzer. Serum CRP concentration was measured by the turbidimetric latex agglutination method (C-Reactive Protein-Latex, Cobas 6000 Roche Diagnostics GmbH, Mannheim, Germany) with a detection limit of 1.0 mg/L. The serum concentration of fibrin degradation products (D-dimer) was measured by the latex agglutination method (D-DI Liatest, Diagnostica Stago, Asnières, France) with a limit of detection of 0.22 *μ*g/mL.

### 2.2. Statistical Analysis

Statistical analysis was performed using software package (Statistica, version 9.0 software package) and data presented as mean values ± SE. Kruskall-Wallis and Mann-Whitney *U* tests were used for comparisons between groups. Correlations between variables were tested using Spearman's test. *P* values less than 0.05 were considered statistically significant.

## 3. Results

Characteristics of the 56 children with acute urticaria and the 41 healthy control subjects is presented in Table 1. Both groups were similar in age (*P* = ns). Positive prick skin test and/or elevated serum sIgE level had 14 children. In children with urticaria serum level of CRP was significantly higher than in the control group. Serum IgE levels in children with single episode of urticaria was higher than in healthy controls.

### 3.1. Serum Levels of IL-1*β*, IL-1RA, and IL-18 in Children with Urticaria and Control Subjects

Serum levels of IL-1*β*, IL-1RA, and IL-18 in children with urticaria and healthy control are shown in Table 2. Serum level of IL-1*β* did not differ between children with acute urticaria (single and recurrent episode) and healthy controls. Children with single episode of urticaria had significantly higher levels of IL-1RA than healthy controls (*P* < 0.004). Serum level of IL-18 in children with single episode of urticaria was significantly higher than in healthy controls and children with recurrent episodes of urticaria (for both *P* < 0.0005). In the group with single episode of acute urticaria, in children with symptoms suggestive of upper respiratory infection (*n* = 21, 53.9%) both serum IL-1RA and IL-18 was significantly higher than in healthy children (*P* < 0.0001, *P* < 0.00008, resp.). The former was also higher than in noninfected urticaria children (*n* = 18, 29.4%) (*P* < 0.01) (Table 2).

Moreover, in all infected urticaria children (single episode and recurrence urticaria, *n* = 26) serum levels of IL-1RA and IL-18 were significantly higher than in healthy controls (*P* < 0.0004, *P* < 0.001, resp.) (data not shown).

The serum concentrations of all examined cytokines in children with urticaria and without infection (single episode and recurrence) did not differ as compared to control group.

### 3.2. Correlation between Serum Levels of IL-1*β*, IL-1RA, IL-18, and D-Dimer and Inflammatory Markers in Children with Single Episode and Recurrent Urticaria

**Figure 1:**
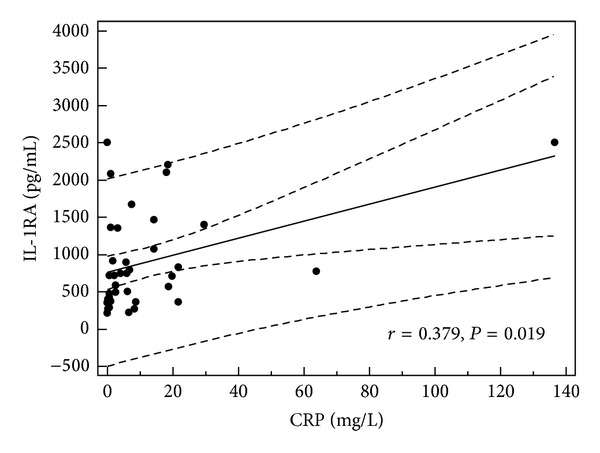
Correlation between serum level of IL-1RA and CRP in children with single episode of acute urticaria.

**Figure 2:**
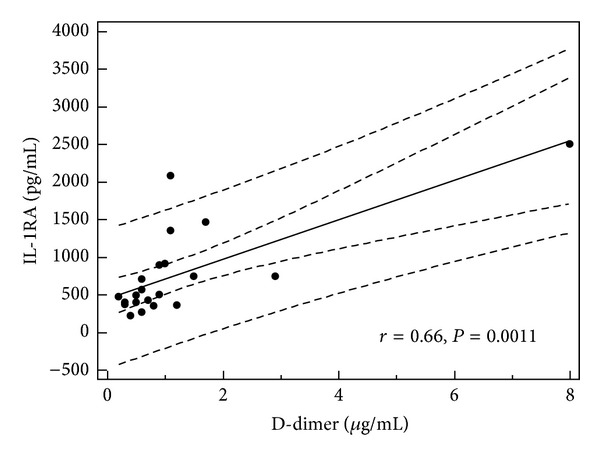
Correlation between IL-1RA and D-dimer in children with single episode of urticaria (all children *n* = 39).

**Figure 3:**
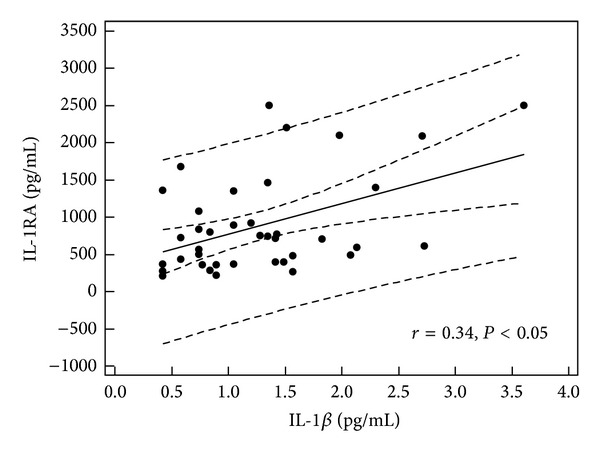
Correlation between IL-1RA and IL-1*β* in children with single episode of urticaria (all children, *n* = 39).

**Figure 4:**
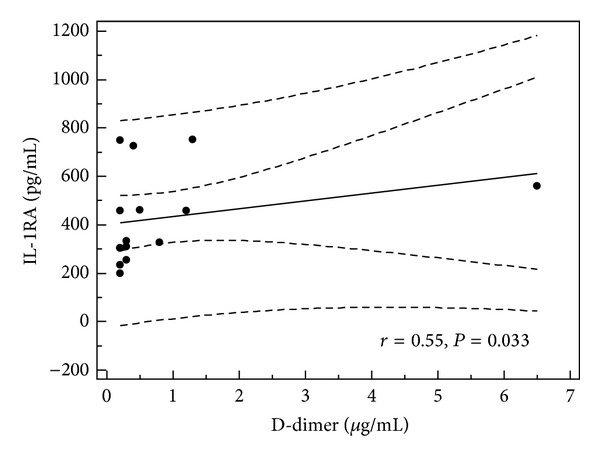
Correlation between serum levels of IL-1RA and D-dimer in children with recurrence of acute urticaria (all children, *n* = 18).

**Figure 5:**
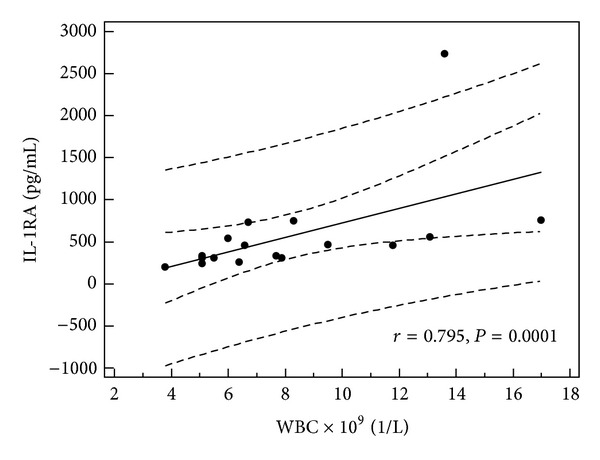
Correlation between serum level of IL-1RA and WBC in children with recurrent urticaria (all children, *n* = 18).

**Figure 6:**
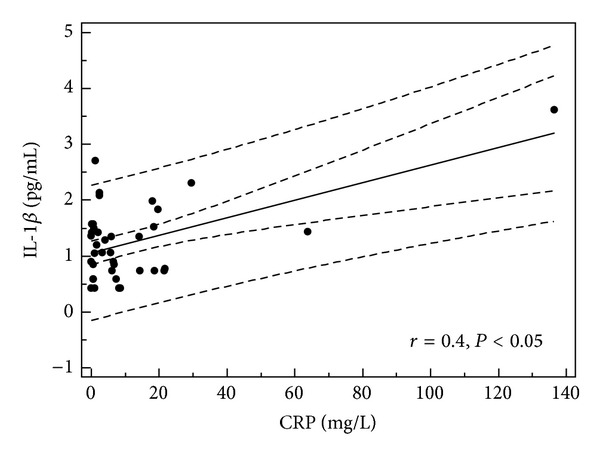
Correlation between serum IL-1*β* level and CPR concentration in children with single episode of acute urticaria (all children, *n* = 39).

**Figure 7:**
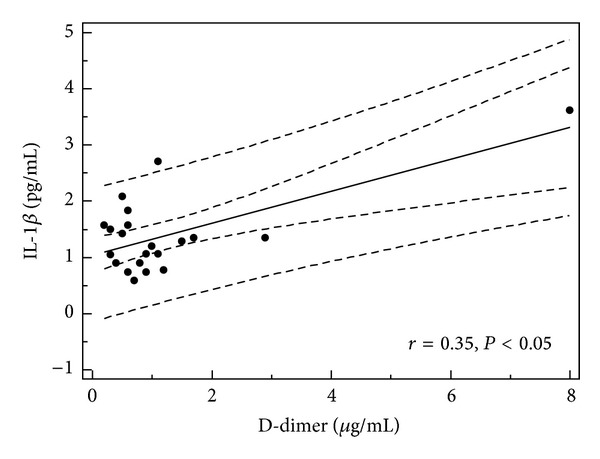
Correlation between serum IL-1*β* level and D-dimer level in children with single episode of acute urticaria (all children, *n* = 39).

In children with single episode of urticaria serum concentration of IL-1RA correlated with CRP, D-dimer, and IL-1*β* levels (*r* = 0.379, *P* = 0.019; *r* = 0.66, *P* = 0.001; and *r* = 0.34, *P* < 0.05, resp.) (Figures 1, 2, and 3). In addition, in children with recurrence of urticaria IL-1RA level correlated positively with D-dimer level and WBC (*r* = 0.55, *P* = 0.033, *r* = 0.79, and *P* = 0.0001) (Figures 4 and 5). Moreover, correlation between serum IL-1*β* level and CPR and D-dimer levels in children with single episode of urticaria was found (*r* = 0.4, *P* < 0.05; *r* = 0.35, *P* < 0.05, resp.) (Figures 6 and 7). No correlation between serum cytokine levels and UAS in children with single episode and recurrent urticaria was observed. In children with single episode of urticaria UAS correlated with CRP levels (*r* = 0.43; *P* < 0.005). We did not find any correlation between examined cytokines and CRP and WBC in control group.

### 3.3. Correlation between Serum Levels of IL-1*β*, IL-1RA, IL-18, and D-Dimer and Inflammatory Markers in Infected and Noninfected Children with Urticaria

Children with single episode of urticaria and upper respiratory tract infection showed a positive correlation of IL-1RA concentration with D-dimer level (*r* = 0.6; *P* < 0.04). Children with recurrent urticaria and infection revealed a positive correlation of IL-1RA level with WBC and CRP concentration (*r* = 0.9; *P* < 0.03, for both). In contrast, in children with single episode of urticaria without infection a positive correlation between IL-1*β* serum level and WBC was found (*r* = 0.5; *P* < 0.02). In addition, children with recurrent urticaria without infection presented a positive correlation of IL-1RA concentration with WBC and IL-1*β* level (*r* = 0.7, *P* < 0.002; *r* = 0.8, *P* < 0.01, resp.) and between IL-1*β* and IL-18 levels (*r* = 0.57 and *P* < 0.05).

## 4. Discussion

The present study was designed to evaluate the circulating levels of IL-1*β*, its receptor IL-1RA and IL-18 in children with single episode, and recurrence of acute urticaria. As could have been expected, IL-1*β* level in serum samples was low and did not differ between children with urticaria and healthy controls. Previous studies, which measured the serum concentration of IL-1*β*, have also revealed that its level in circulation is usually very low, even in septic shock patients [30]. Moreover, no concentration of circulating IL-1*β* was detected in patients with inherited chronic autoinflammatory syndromes, but increased secretion of IL-1*β* was observed *in vitro* in such a condition [20]. Interestingly, in CAPS, in which increased secretion of IL-1*β* is due to a single nucleotide mutation in the *NALP3* gene, controlling the caspase-1 activity, the main source of IL-1, are the mast cells [23].

In our study, despite low levels, in children with single episode of urticaria IL-1*β* correlated with CRP and D-dimer levels (marker of coagulation pathway activation). However, recent studies have documented that overproduction of IL-1 is important in inflammation because it induces IL-6 excretion, accounts for hepatic acute-phase protein synthesis (CRP), and contributes to transendothelial passage of neutrophils by increasing the surface expression of endothelial adhesion molecules and production of IL-8 [14, 30, 31]. IL-1*β* appears to play a crucial role in the initiation of coagulation and fibrinolysis [32]. Several of the latest studies focusing on the relationship between the levels of coagulation and inflammation markers with the acute phase exacerbation indicators and the degree of severity of chronic urticaria have been conducted [33–35]. It was shown that D-dimer was closely related to the activation of urticaria as well as CRP levels [32, 34].

The potent biological effects of IL-1*β* are negatively regulated by secretory interleukin-1 receptor antagonist (sIL-1RA). The secretory IL-1RA expression can be induced by proinflammatory stimuli such as IL-1*β* itself, viruses, bacteria, and microbial products, as well as a variety of immunomodulators such as GMCSF, IFN-*β*, and IFN-*γ*. As mentioned above, serum concentration of IL-*β* is usually low, and in such cases IL-1RA is measured as an indicator of the severity of various infections and noninfectious diseases [28]. A significant increase of IL-1RA was observed during experimental endotoxemia in human, in a variety of inflammatory, infectious, cardiovascular, and postsurgical conditions, indicating the importance of this anti-inflammatory protein [36–38]. The regulation of expression of IL-1RA might play a key role in termination of an immune-mediated inflammatory response. Therapy with the recombinant IL-1 receptor antagonist, such as anakira, results in complete remission in patients with neutrophilic urticaria and autoinflammatory disorder such as CAPS, FCAS (familial cold autoinflammatory syndrome), and DIRA (deficiency of interleukin-1 receptor antagonist) [23, 39].

In our study, the serum level of IL-1RA was elevated in children with acute urticaria and evidence of infection, likely representing a protective response to rise of IL-1*β*. Moreover, IL-1RA level was correlated with IL-1*β* level, CRP, and D-dimer levels in children with single episode of urticaria and WBC and D-dimer concentration in children with the recurrence of urticaria.

We documented that IL-1*β* and IL-1RA level in infected and noninfected children with urticaria correlate differently with D-dimer and the markers of inflammation including CRP and WBC. Due to the small number of subjects in the groups the evaluation requires further continuation.

We have also showed that serum level of IL-18 in children with single episode of urticaria and symptoms of upper respiratory infection was increased as compared to healthy subjects. Since IL-18 contributes to host defense against invading pathogens [14] we could not exclude that elevation of IL-18 similarly to IL-1RA in examined children is an early marker of acute phase of infection. On the other hand, this proinflammatory cytokine may play some role in the pathogenesis of acute urticaria possibly through its capacity to stimulate histamine release by basophils [12]. IL-18 also upregulates expression of IL-8 by eosinophils and enhances recruitment of neutrophils to inflamed tissues [40].

There are several reports that IL-18 plays a pathogenically important role in chronic inflammatory conditions of epithelial organs (such as skin, gut, and kidney) and allergic diseases [17, 27]. Higher serum levels of IL-18 have previously been identified in atopic dermatitis and asthmatic subjects [17, 41]. An elevated level of IL-18 is a prominent feature of Schnitzler syndrome, a rare disorder characterized by chronic urticarial rash and monoclonal gammopathy, along with fever, arthralgia, or bone pain [23]. Overproduction of IL-18 expression was described in some adults with chronic spontaneous urticaria [42, 43] but not in all studies [25].

In conclusion, in this preliminary study in children we give evidence that serum IL-1RA and IL-18 levels are increased in some children with acute urticaria. However, further studies should be conducted on a large population to define a possible pathogenic role of IL-1*β*, its antagonist receptor IL-1RA and IL-18 in urticaria.

## Figures and Tables

**Table 1:** Demographic and clinical characteristics of children with urticaria and healthy children.

	Acute urticaria—single episode	Recurrent urticaria	Healthy children
Number	39	17	41
Age (y.)	8.56 ± 1.67	9.61 ± 2.45	9.36 ± 1.57
M/F	16/23	7/10	17/24
CRP (mg/L)	11.99 ± 7.67*	7.44 ± 8.08**	1.05 ± 0.59
WBC (10^9^/L)	9.44 ± 1.21	8.19 ± 1.74	7.3 ± 1.54
D-dimer	1.23 ± 0.71^#^	0.85 ± 0.81^##^	nd
*E* % (absolute count, cells/*µ*L)	1.94 ± 1.94 (236.87 ± 158.7)	1.47 ± 0.95 (136.14 ± 18.13)	1.9 ± 0.96 (177.3 ± 60)
IgE (kU/L)	225.79 ± 102.6	102.82 ± 75.79	72 ± 47.17
IgA (g/L)	1.22 ± 0.17	1.20 ± 0.17	1.13 ± 0.31
IgM (g/L)	1.04 ± 0.09	0.94 ± 0.08	0.99 ± 0.21
IgG (g/L)	8.53 ± 0.59	8.54 ± 0.66	8.63 ± 0.98
Personal allergic disease	9	5	
Family allergic disease	5	8	
Symptoms suggestive of infection	21	5	
UAS 1/2/3^&^	12/10/17	11/3/3	

Data are shown as mean ± standard error. *P* values from Mann-Whitney *U* test.

*Children with single episode of acute urticaria versus control group *P* < 0.00001.

**Children with recurrent urticaria versus control group *P* < 0.007.

^#^ *n* = 22, ^##^ *n* = 15.

^&^Urticaria activity score: 1 mild urticaria (0–3), 2 moderate urticaria (3-4), and 3 severe urticaria (5-6).

**Table 2:** Serum levels of IL-1*β*, IL-1RA, and IL-18 in children with urticaria and healthy children.

Groups	Number	IL-1*β* (pg/mL)	IL-1RA (pg/mL)	IL-18 (pg/mL)
Single episode of acute urticaria				
All children	39	1.3 ± 0.8	889.1 ± 205.3*	98.0 ± 18.7**
Children with signs of infection	21	0.7 ± 0.6	1129.8 ± 676.0^#^	102.0 ± 47.8^##^
Children without infection	18	1.6 ± 0.8	608.3 ± 512.0	93.1 ± 71.3
Recurrent urticaria				
All children	17	1.6 ± 0.9	570.5 ± 278.1	56.6 ± 8.6
Children with signs of infection	5	0.8 ± 0.2	512.7 ± 372.2	54.8 ± 16.4
Children without infection	12	1.9 ± 2.1	594.6 ± 242.5	56.9 ± 19.3
Healthy controls	41	1.3 ± 0.3	529.4 ± 114.8	62.2 ± 7.4

Data are shown as mean ± standard error. *P* values from Mann-Whitney *U* test.

*All children with single episode of acute urticaria versus control group, *P* < 0.004.

**All children with single episode of acute urticaria versus control group and children with recurrent urticaria, *P* < 0.0005.

^#^Patients with single episode of urticaria—children with signs of infection versus children without infection, *P* < 0.01 and versus control group, *P* < 0.0001.

^##^Patients with single episode of urticaria—children with signs of infection versus control group, *P* < 0.00008.
